# Optimizing identification of Lyme disease diagnoses in commercial insurance claims data, United States, 2016–2019

**DOI:** 10.1186/s12879-024-10195-5

**Published:** 2024-11-20

**Authors:** Courtney C. Nawrocki, Austin R. Earley, Sarah A. Hook, Alison F. Hinckley, Kiersten J. Kugeler

**Affiliations:** 1 Division of Vector-Borne Diseases, Centers for Disease Control and Prevention, Fort Collins, CO USA; 2 Oak Ridge Institute for Science and Education, Oak Ridge, TN USA

**Keywords:** Lyme disease, Surveillance, Epidemiology

## Abstract

**Background:**

Commercial insurance claims data are a stable and consistent source of information on Lyme disease diagnoses in the United States and can contribute to our understanding of overall disease burden and the tracking of epidemiological trends. Algorithms consisting of diagnosis codes and antimicrobial treatment information have been used to identify Lyme disease diagnoses in claims data, but there might be opportunity to improve their accuracy.

**Methods:**

We developed three modified versions of our existing claims-based Lyme disease algorithm; each reflected refined criteria regarding antimicrobials prescribed and/or maximum days between diagnosis code and qualifying prescription claim. We applied each to a large national commercial claims database to identify Lyme disease diagnoses during 2016–2019. We then compared characteristics of Lyme disease diagnoses identified by each of the modified algorithms to those identified by our original algorithm to assess differences from expected trends in demographics, seasonality, and geography.

**Results:**

Observed differences in characteristics of patients with diagnoses identified by the three modified algorithms and our original algorithm were minimal, and differences in age and sex, in particular, were small enough that they could have been due to chance. However, one modified algorithm resulted in proportionally more diagnoses in men, during peak summer months, and in high-incidence jurisdictions, more closely reflecting epidemiological trends documented through public health surveillance. This algorithm limited treatment to only first-line recommended antimicrobials and shortened the timeframe between a Lyme disease diagnosis code and qualifying prescription claim.

**Conclusions:**

As compared to our original algorithm, a modified algorithm that limits the antimicrobials prescribed and shortens the timeframe between a diagnosis code and a qualifying prescription claim might more accurately identify Lyme disease diagnoses when utilizing insurance claims data for supplementary, routine identification and monitoring of Lyme disease diagnoses.

**Supplementary Information:**

The online version contains supplementary material available at 10.1186/s12879-024-10195-5.

## Background

Lyme disease, caused by *Borrelia burgdorferi* sensu lato spirochetes and transmitted to humans by *Ixodes* spp. ticks, is the most commonly reported vector-borne disease in the United States, with cases geographically focused in the Northeast, mid-Atlantic, and upper Midwest regions [1, 2]. Lyme disease data are voluntarily reported to the Centers for Disease Control and Prevention (CDC) by state and local public health agencies via the National Notifiable Disease Surveillance System (NNDSS) [3]. During 2008–2021, 30,000–40,000 cases of Lyme disease were typically reported each year; trends consistently indicated a peak in the summer months, a slight predominance in men, and a bimodal age distribution, with the highest rates among younger children and older adults [1, 4].

Historically, substantial underreporting of Lyme disease cases has occurred, most commonly in highly endemic areas where the case burden is greatest and significant human resources are required by health departments to investigate cases [5–7]. Additionally, while the case definition used for public health surveillance for Lyme disease is specific and meant to identify high-risk groups and track disease trends and geographic distribution over time, it does not capture all Lyme disease cases in the United States [8]. Alternative data sources are needed to improve understanding of the true burden of Lyme disease and to allow for supplementary monitoring of epidemiological trends [9].

Insurance claims databases have been used as an alternative data source for identifying Lyme disease diagnoses as they contain standardized diagnosis codes, information on prescription medication claims and, in some cases, laboratory test orders [9–23]. Our group previously evaluated the utility of a national commercial claims database for routine identification of Lyme disease diagnoses and found that during 2010–2018, the estimated annual incidence was 6–8 times as high as that observed for cases reported through public health surveillance [12]. This study used an algorithm initially developed to maximize sensitivity for identifying Lyme disease diagnoses in claims data, first described in Nelson et al. [11]; it defined an outpatient Lyme disease diagnosis as the first outpatient encounter in a calendar year with an International Classification of Diseases (ICD) diagnosis code for Lyme disease and a prescription claim for ≥ 7 days of a qualifying antimicrobial drug within ± 30 days of the encounter date.

Schwartz et al. [12] found that while age and sex distributions for identified Lyme disease diagnoses were similar to those demonstrated in public health surveillance, more diagnoses occurred outside of typical peak summer months, among females, and in low-incidence jurisdictions compared to cases reported through surveillance, indicating suboptimal specificity of this algorithm. Another study assessed the validity of the algorithm against medical chart review and found high (93.8%) positive predictive value (PPV) for identifying confirmed, probable, or suspect cases of Lyme disease but a somewhat lower PPV (66.4%) for identifying confirmed and probable cases [24]. This study was unable to calculate the algorithm’s specificity.

We saw two opportunities to potentially improve the accuracy of this established claims-based algorithm for identifying Lyme disease diagnoses. First, the list of qualifying antimicrobial drugs for Lyme disease was broad; in addition to first-line recommended therapies, it included second-line therapies and some drugs that are no longer recommended for the treatment of Lyme disease [25]. Second, it established a broad, 30-day allowable time window for a prescription claim around an ICD code for Lyme disease, which could potentially lead to capture of prescription claims unrelated to the Lyme disease ICD code.

We sought to develop and evaluate three modified versions of our existing treatment-based Lyme disease algorithm [11, 12], intended to improve accuracy by restricting to first-line recommended antimicrobials and reducing the allowable time window between an ICD code and a prescription claim. We compared characteristics of identified diagnoses to those identified by the original algorithm and to epidemiological trends consistently seen with public health surveillance. Our ultimate goal was to optimize our claims-based algorithm for routine tracking of Lyme disease diagnoses using this data source.

## Methods

### Data source

We used data from the Merative MarketScan Commercial Claims and Encounters Databases [26], which contains deidentified insurance claims records for > 25 million privately insured individuals aged < 65 years annually for inpatient, outpatient, and drug prescription services. The MarketScan database is demographically similar to the U.S. population aged < 65 years and has been found to be a stable source of data for routine analyses of epidemiologic trends in Lyme disease diagnoses [12]. For this analysis, we restricted the MarketScan population to a cohort of outpatient enrollees with insurance coverage for the entire calendar year each year during 2016–2019.

### Identification of Lyme disease diagnoses

We developed three modified versions of our previously developed algorithm (hereafter referred to as the original algorithm (OA)) [11, 12], which includes ICD codes for Lyme disease and prescription claims for appropriate antimicrobials consistent with established treatment recommendations during 2016–2019 (hereafter referred to as qualifying prescription claims) (Table 1) [27]. We did not include laboratory data in our algorithm as it is available for only a small subset of enrollees in the MarketScan database and would greatly limit our sample size.

**Table 1:** Criteria for algorithms used to identify Lyme disease diagnoses in MarketScan database, 2016–2019

Algorithm name	Criteria
Original Algorithm (OA)^a^	ICD-10-CM^b^ code A69.2x AND a prescription claim for ≥ 7 days treatment with any appropriate antimicrobial drug^c^ within ± 30 days.
Modified Algorithm (MA) 1	ICD-10-CM code A69.2x AND a prescription claim for ≥ 7 days treatment with a first-line antimicrobial drug^b^ within ± 30 days.
Modified Algorithm (MA) 2	ICD-10-CM code A69.2x AND a prescription claim for ≥ 7 days treatment with any appropriate antimicrobial drug^b^ within ± 14 days.
Modified Algorithm (MA) 3	ICD-10-CM code A69.2x AND a prescription claim for ≥ 7 days treatment with a first-line antimicrobial drug^b^ within ± 14 days.

^a^ [11, 12]

^b^International Classification of Diseases, 10th Revision, Clinical Modification

^c^In accordance with the established guidelines during the study period [27]

We applied each of the three modified algorithms (MAs) and the OA to the 2016–2019 MarketScan cohort. For each algorithm, we retained a patient’s first Lyme disease diagnosis in a calendar year (i.e., first encounter where an ICD code for Lyme disease was assigned with a qualifying prescription claim within the specified timeframe); patients could have more than one Lyme disease diagnosis in the four-year study period but only one diagnosis per calendar year. For patients who had multiple qualifying prescription claims within the specified timeframe around the encounter when an ICD code was assigned, we retained only the prescription claim that occurred closest in time to (before or after) the encounter date. When more than one qualifying prescription claim occurred within the same number of days from the ICD code, we prioritized by line of therapy in accordance with the established treatment recommendations, retaining only the drug of the highest line of therapy [27]. When more than one prescription claim for drugs of the same line of therapy occurred within the same number of days from the ICD code, we retained the drug that came first in alphabetical order based on its generic name to simplify data management and analysis.

### Comparison of Lyme disease diagnoses identified by modified algorithms

We compared the number of Lyme disease diagnoses identified by each of the MAs and the relative proportions of their characteristics to those identified by the OA and to trends demonstrated through public health surveillance. Characteristics compared were age, sex, seasonality, and geographic risk level. Geographic risk level was assigned based on annual average incidence of a patient’s jurisdiction of residence between 2016 and 2019; high-incidence jurisdictions included those with ≥ 10 confirmed cases of Lyme disease per 100,000 population during the reporting period, and all other jurisdictions were classified as either unknown incidence or low incidence (Appendix A). Patients with unknown jurisdiction of residence but who were assigned a geographic region of “Northeast” were assigned to the high-incidence category because all states or jurisdictions included in this region were considered high incidence during the study period. Patients who were assigned a state of residence of “New England Division, unknown state” or “Middle Atlantic Division, unknown state” were also included in the high-incidence category (Appendix A). To assess seasonality, month of Lyme disease diagnosis was defined by the date of the encounter when the ICD code was assigned.

Additionally, we calculated the mean and standard deviation (SD)), median, and inter quartile range (IQR) of the number of days between assignment of an ICD code for Lyme disease and a prescription claim for a qualifying antimicrobial and compared by algorithm and drug. All analyses were conducted in R version 4.0.2 and SAS version 9.4 [28, 29].

## Results

During 2016–2019, we identified a total of 55,661 Lyme disease diagnoses using the OA; 50,052 Lyme disease diagnoses using MA 1, which restricted qualifying prescription claims to only first-line recommended antimicrobial drugs; 50,617 Lyme disease diagnoses using MA 2, which restricted the timeframe between an ICD code and qualifying prescription claim to ± 14 days; and 45,878 Lyme disease diagnoses using MA 3, which combined MA 1 and MA 2, restricting qualifying prescription claims to only first-line recommended antimicrobial drugs and the timeframe between an ICD code and prescription claim to ± 14 days. For all four algorithms, 2017 had the highest number of Lyme disease diagnoses and 2019 had the lowest (Table 2).

**Table 2:** Characteristics among Lyme disease diagnoses identified by algorithms in MarketScan database, 2016–2019

Characteristic	OA^a, b^ *N* = 55,661		MA^c^ 1^d^ *N* = 50,052		MA 2^e^ *N* = 50,617		MA 3^f^ *N* = 45,878	
	No.	(%)	No.	(%)	No.	(%)	No.	(%)
Year								
2016	14,243	25.6	12,789	25.6	12,989	25.7	11,746	25.6
2017	15,890	28.5	14,372	28.7	14,495	28.6	13,201	28.8
2018	13,275	23.8	11,900	23.8	12,082	23.9	10,932	23.8
2019	12,253	22.0	10,991	22.0	11,051	21.8	9,999	21.8
Sex								
Male	27,157	48.8	25,146	50.2	25,030	49.5	23,335	50.9
Female	28,504	51.2	24,906	49.8	25,587	50.5	22,543	49.1
Age Group (years)								
0–17	11,386	20.5	10,368	20.7	10,682	21.1	9,784	21.3
18–34	9,693	17.4	8,645	17.3	8,779	17.3	7,877	17.2
35–44	8,327	14.9	7,388	14.8	7,428	14.7	6,643	14.5
45–54	12,402	22.3	10,961	21.9	11,200	22.1	10,005	21.8
55–64	13,853	24.9	12,690	25.3	12,528	24.8	11,569	25.2
Geographic Risk Level^g^								
High incidence	40,917	73.5	37,440	74.8	37,420	73.9	34,502	75.2
Low incidence	10,410	18.7	8,679	17.3	9,282	18.4	7,779	17.0
Unknown incidence	4,334	7.8	3,933	7.9	3,915	7.7	3,597	7.8
Summer Diagnosis^h^								
Yes	31,519	56.6	29,516	59.0	29,343	58.0	27,636	60.2
No	24,142	43.4	20,536	41.0	21,274	42.0	18,242	39.8

^a^Original Algorithm

^b^ICD-10-CM code A69.2x AND a prescription claim for ≥ 7 days treatment with any appropriate antimicrobial drug^b^ within ± 30 days

^c^Modified Algorithm

^d^ICD-10-CM code A69.2x AND a prescription claim for ≥ 7 days treatment with a first-line antimicrobial drug^b^ within ± 30 days

^e^ICD-10-CM code A69.2x AND a prescription claim for ≥ 7 days treatment with any appropriate antimicrobial drug^b^ within ± 14 days

^f^ICD-10-CM code A69.2x AND a prescription claim for ≥ 7 days treatment with a first-line antimicrobial drug^b^ within ± 14 days

^g^Based on patient’s state of residence

^h^Diagnosis code was assigned in the months of May, June, July, or August

### Comparison of characteristics of Lyme disease diagnoses by algorithm

**Fig. 1:**
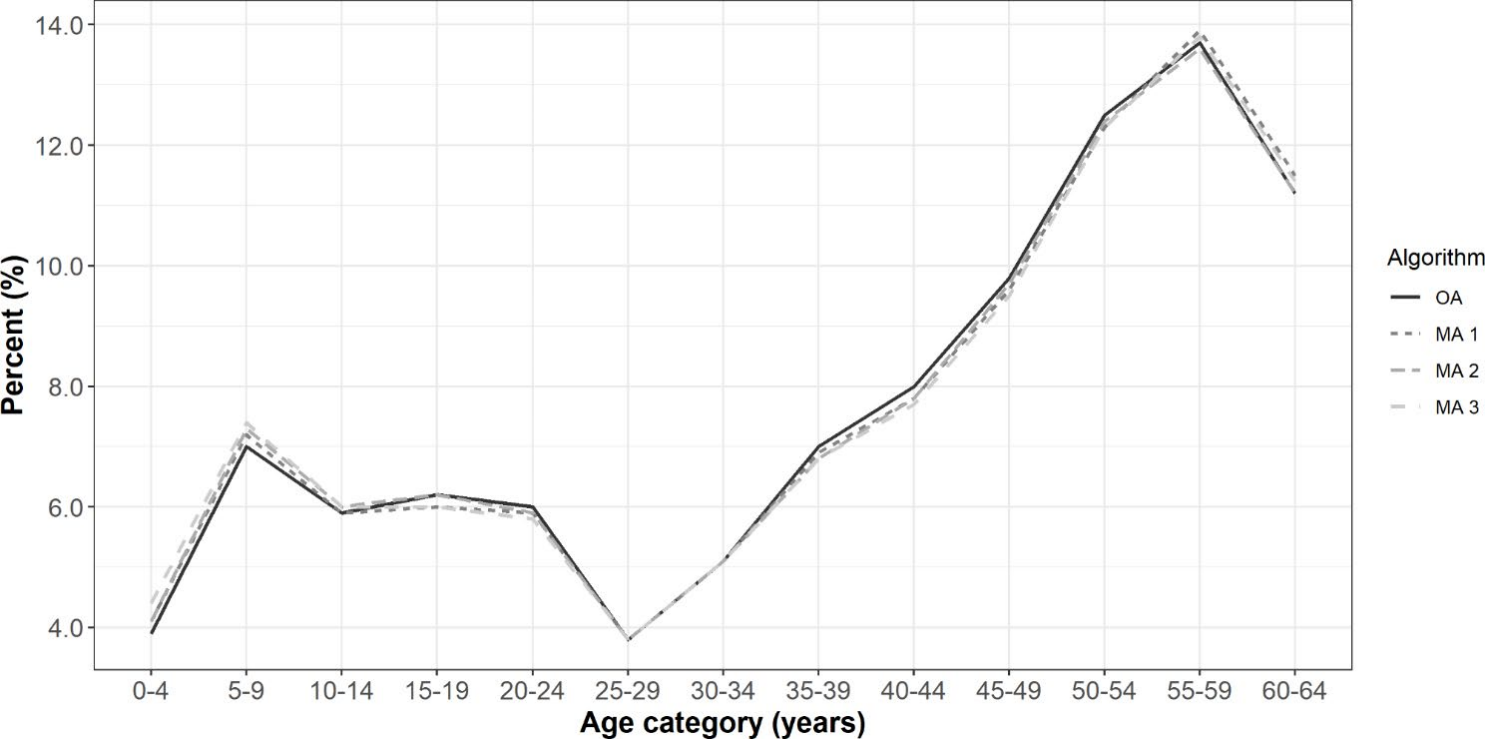
Age distribution among Lyme disease diagnoses identified by algorithms in MarketScan database, 2016–2019. **Legend**: OA = Original Algorithm; MA 1 = Modified Algorithm 1; MA 2 = Modified Algorithm 2; MA 3 = Modified Algorithm 3

Overall, characteristics of Lyme disease diagnoses did not differ greatly between the three modified case definitions and the OA, and thus it is possible that observed differences were due to chance. We did observe a slight male predominance among diagnoses identified by MA 1 and MA 3, and a slight female predominance among diagnoses identified by MA 2 and the OA (Table 2). MA 3 had the highest proportion of diagnoses in the 5-9-year age group (7.4%) compared to the OA, which had the lowest (7.0%), and MA 1 had the highest proportion of diagnoses in the 55-59-year age group (13.9%) compared to MA 2, which had the lowest (13.6%) (Fig. 1).

**Fig. 2:**
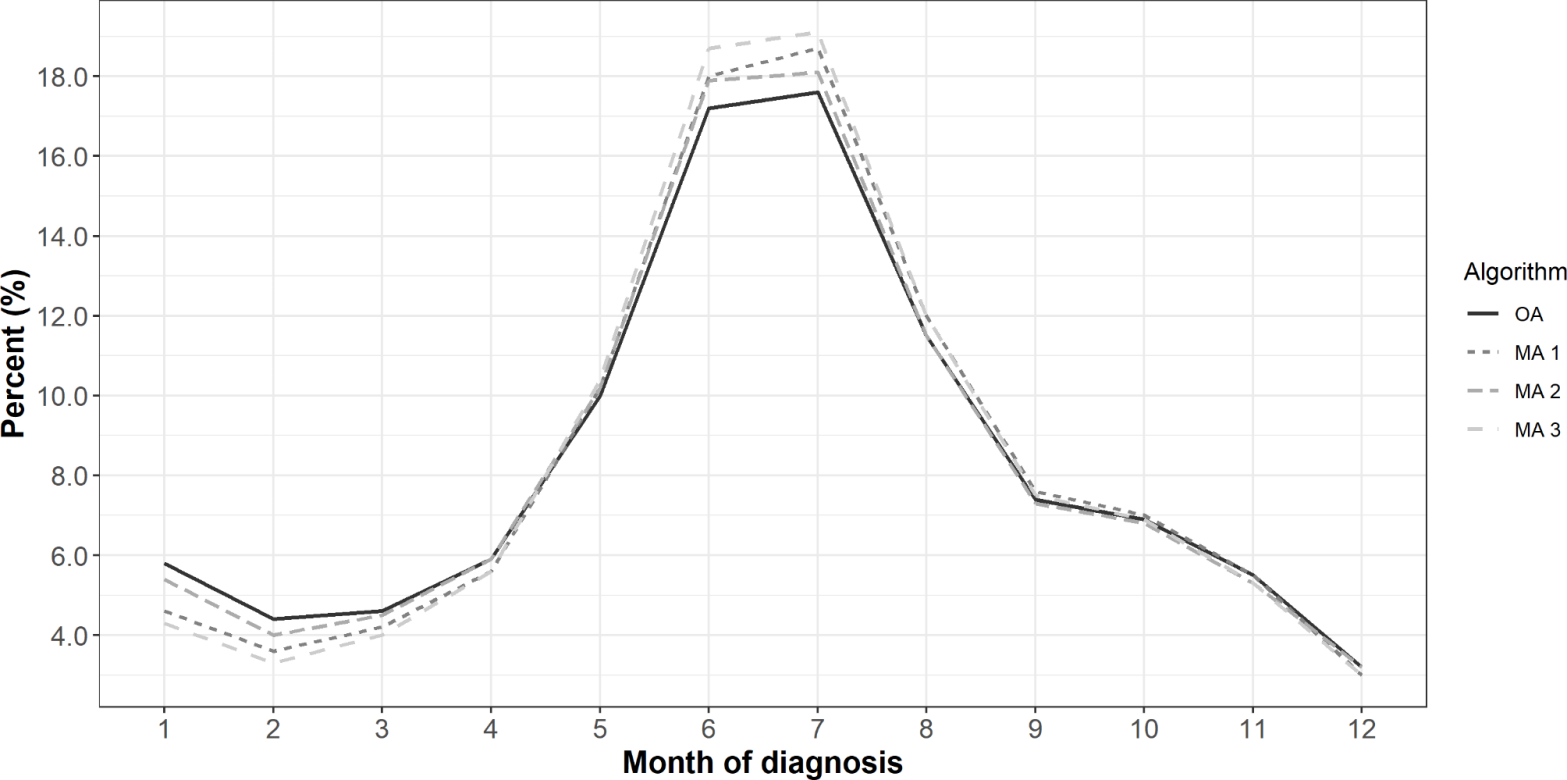
Seasonality of Lyme diagnoses identified by algorithms in MarketScan database, 2016–2019. **Legend**: OA = Original Algorithm; MA 1 = Modified Algorithm 1; MA 2 = Modified Algorithm 2; MA 3 = Modified Algorithm 3. Month of diagnosis refers to the month that the ICD code was assigned

All three MAs had a higher proportion of diagnoses in high-incidence jurisdictions compared to the OA, with MA 3 having the highest, followed by MA 1 and MA 2 (Table 2). All three MAs also had a higher proportion of diagnoses during the months of May-August compared to the OA, with MA 3 being the highest, followed by MA 1 and MA 2 (Fig. 2). The majority of diagnoses during the months of May-August were among men, with 52.0% identified by the OA, 52.5% identified by MA 2, 52.9% identified by MA 1, and 53.4% identified by MA 3.

### Mean number of days between a Lyme disease diagnosis code and a qualifying prescription claim

The overall mean number of days from an ICD code encounter to the closest qualifying prescription claim was less than one day for all four algorithms (MA 1: -0.27 ± 8.53 (SD); MA 2: 0.14 ± 4.41 (SD); MA 3: 0.06 ± 4.16 (SD); OA: -0.05 ± 9.01 (SD)). The median and IQR were both zero for all four algorithms.

**Fig. 3:**
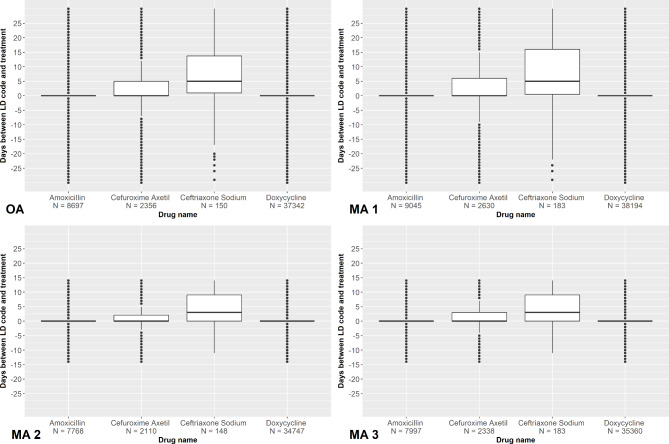
Days from diagnosis code to prescription claim among Lyme disease diagnoses in MarketScan database, 2016–2019. **Legend**: This plot displays data for only first-line drugs for all four algorithms, including MA 2 and OA, which, by definition, do not restrict qualifying drugs to first-line only. MA 1 = Modified Algorithm 1; MA 2 = Modified Algorithm 2; MA 3 = Modified Algorithm 3; OA = Original Algorithm. OA: Amoxicillin (Mean ± SD: 0.2 ± 9.0, Median: 0, IQR: 0); Cefuroxime axetil (Mean ± SD: 1.7 ± 10.2, Median: 0, IQR: 5), Ceftriaxone sodium (Mean: 6.5 ± 10.8, Median: 5, IQR: 12.8); Doxycycline (Mean ± SD: -0.7 ± 7.7, Median: 0, IQR: 0). MA 1: Amoxicillin (Mean ± SD: 0.3 ± 9.5, Median: 0, IQR: 0); Cefuroxime axetil (Mean ± SD: 2.2 ± 10.9, Median: 0, IQR: 6); Ceftriaxone sodium (Mean ± SD: 7.3 ± 11.8, Median: 5, IQR: 15.5); Doxycycline (Mean ± SD: -0.6 ± 8.0, Median: 0, IQR: 0). MA 2: Amoxicillin (Mean ± SD: 0.1 ± 4.0, Median: 0, IQR: 0); Cefuroxime axetil (Mean ± SD: 1.1 ± 5.1, Median: 0, IQR: 2); Ceftriaxone sodium (Mean ± SD: 4.1 ± 5.8, Median: 3, IQR: 9); Doxycycline (Mean ± SD: -0.1 ± 3.9, Median: 0, IQR: 0). MA 3: Amoxicillin (Mean ± SD: 0.2 ± 4.2, Median: 0, IQR: 0); Cefuroxime axetil (Mean ± SD: 1.2 ± 5.3, Median: 0, IQR: 3); Ceftriaxone sodium (Mean ± SD: 4.0 ± 6.1, Median: 3, IQR: 9); Doxycycline (Mean ± SD: -0.1 ± 4.0, Median: 0, IQR: 0)

When looking at the qualifying prescription claim that occurred closest in time to a patient’s ICD code encounter, doxycycline was the most commonly prescribed drug across all four algorithms (range 34,747 − 38,194 prescriptions) and had the lowest mean number of days from ICD code assignment to prescription claim among all qualifying drugs (Fig. 3 and Appendix B). Among MA 1 and MA 3, which limited qualifying drugs to first-line therapies only, ceftriaxone sodium was the least common drug to occur closest in time to a patient’s ICD code encounter (183 prescriptions for both MA 1 and MA 3), and among MA 2 and the OA, which did not limit qualifying drugs to first-line therapies only, erythromycin was the least commonly prescribed drug to occur closest in time to a patient’s ICD code encounter (52 and 60 prescriptions, respectively). Overall, ceftriaxone sodium was the drug with the highest mean number of days from ICD code assignment to prescription claim when it was the drug prescribed closest in time to a patient’s ICD code encounter (Fig. 3 and Appendix B).

## Discussion

In this study, we sought to improve our confidence in the accuracy of a claims-based algorithm for Lyme disease by applying and comparing diagnoses resulting from three different modifications to our existing algorithm: (1) restriction to prescription claims for first-line recommended antimicrobials for Lyme disease only (MA 1), (2) reduction of the amount of time between a diagnosis code for Lyme disease and a qualifying prescription claim from ± 30 days to ± 14 days (MA 2), and (3) a combination of both of these modifications (MA 3). While observed differences in characteristics of diagnoses identified by each algorithm, particularly age and sex, were small enough that they could have been artifacts of random data variability, MA 3 resulted in the most diagnoses in men, high-incidence jurisdictions, and summer months compared to the other algorithms, rendering it the most consistent with trends observed in public health surveillance for Lyme disease.

The slight female predominance in diagnoses identified by MA 2 and the OA and less pronounced peak in diagnoses among those 5–9 years of age is consistent with results previously demonstrated by the OA in Schwartz et al. [12]; other studies that utilized claims data to identify Lyme disease diagnoses also observed a slight female predominance [9, 10]. However, one study that looked at Medicaid claims data found that males were significantly more likely to be diagnosed with Lyme disease during the months of June-August and less likely to be diagnosed during the months of December-March compared to females [19]. This aligns with our finding that the majority of diagnoses in the months of May-August were among men, with MA 3 and MA 1 having the highest proportions, though differences were small.

Notably, the overall mean number of days from Lyme disease ICD code assignment to a prescription claim were negative for MA 1 and the OA, and when we limited to prescription claims for doxycycline only, it was negative across all four algorithms. This indicates that the prescription claim occurred before an ICD code for Lyme disease was assigned and could be reflective of coding practices, such as cases where a code might have been applied retrospectively. It could also reflect presumptive treatment of Lyme disease by a clinician before an in-person visit could occur, such as in the case of telehealth visits, which we did not specifically explore.

Diagnostic coding practices are known to vary [30, 31], and ICD codes that occur in proximity to a prescription claim for an appropriate antimicrobial could be unrelated. For example, an ICD code may linger in a patient’s record from a previous diagnosis or represent clinician suspicion or differential diagnosis. Additionally, some of the second-line antimicrobials included in the OA and MA 3 algorithms are infrequently used to treat Lyme disease and no longer recommended [25]. The steps we took to restrict to first-line recommended antimicrobials and limit the timeframe between occurrence of an ICD code and a qualifying prescription claim reduces the potential that our algorithm is identifying these scenarios as incident Lyme disease diagnoses, increasing our overall confidence in the validity of diagnoses identified.

While Lyme disease diagnoses identified by MA 3 displayed trends most consistent with public health surveillance data, trends in diagnoses identified by all four algorithms generally reflected those seen in national surveillance data, and the close temporal distribution of prescription claims for first-line recommended antimicrobials around the day an ICD code for Lyme disease was assigned lends confidence to the use of these algorithms for supplemental, routine tracking of Lyme disease diagnoses in insurance claims data.

Cocoros et al. [24], calculated PPV of the same claims-based algorithm (the OA) that we propose modifications to here. While PPV is a measure particularly useful in clinical settings, we took steps intended to increase the overall accuracy of a claims-based algorithm, recognizing that while Lyme disease diagnoses identified in claims data represent only a fraction of true Lyme disease cases, they are a measurable and trackable fraction. We echo the sentiments of other authors that further efforts are needed to directly assess performance of our proposed modified algorithms and other algorithms developed for Lyme disease diagnosis identification and tracking in claims data [32].

### Limitations

Results generated using claims-based case definitions may not be representative of the general population; while MarketScan is a large commercial insurance claims database and has been determined to be geographically representative and demographically similar to the U.S. population [12], it does not contain information for persons who are uninsured, ≥ 65 years of age, or military personnel, impacting generalizability. However, the trends identified in this study are similar to those seen in other studies that utilized insurance claims-based systems, which lends confidence to our findings. Additionally, claims-based algorithms rely on diagnosis codes, which are often under-captured in administrative claims data and subject to error from the clinicians and billing specialists who assign them. This could lead to misclassification and the potential for both overreporting and underreporting of Lyme disease diagnoses.

Because of variations in coding practices, it is also possible that some patients identified as having Lyme diseases diagnoses by our algorithms received treatment for another condition unrelated to the ICD code or were treated empirically; thus, diagnoses, as defined here, do not necessarily equate to true infection with *B. burgdorferi*. Additionally, to simplify our database query, our algorithms required a minimum of seven days of treatment with a qualifying antimicrobial, but established guidelines at the time recommended a minimum duration longer than seven days for some qualifying antimicrobials, increasing the potential of capturing diagnoses that were not actually Lyme disease. We attempted to limit this through our modifications to restrict to first-line recommended antimicrobials only and by shortening the timeframe between an ICD code for Lyme disease and a prescription claim. The finding that treatment was prescribed, on average, less than one day from when an ICD code for Lyme disease was assigned for all four algorithms further increased our confidence that we are accurately capturing treatments prescribed for Lyme disease.

A small proportion of patients had prescription claims for more than one first-line recommended antimicrobial on the same day (OA: 0.5%, MA 1: 0.6%, MA 2: 0.5%, MA 3: 0.6%). Because we retained only the name of the generic drug that came first in alphabetical order in these cases, the antimicrobial that we categorized these patients as having received to treat a Lyme disease diagnosis may be misclassified or skewed towards generic drug names that come earlier in the alphabet. However, doxycycline, the most commonly recommended first line drug for treatment of Lyme disease [27], was by far the most commonly prescribed drug in prescription claims across all four algorithms, despite coming last in alphabetical order among the first-line drugs.

Lastly, by dichotomizing geographical heterogeneity of Lyme disease risk into high and low incidence, we did not separately describe the frequency or characteristics of Lyme disease diagnoses among patients living in jurisdictions with emerging Lyme disease incidence, particularly those that border high-incidence jurisdictions. Thus, the frequency of Lyme disease diagnoses in low-incidence states might be inflated due to inclusion of diagnoses from areas with emerging risk.

## Conclusions

Modification of our claims-based algorithm for Lyme disease to limit to only first-line recommended antimicrobials and shorten the timeframe between a Lyme disease diagnosis code and a prescription claim to ± 14 days might improve accuracy of identification of Lyme disease diagnoses. This modified algorithm may be useful for other investigators in identifying and monitoring Lyme disease diagnoses in both commercial claims and health-system-based administrative databases; standardized use of such an algorithm could facilitate comparisons across studies.

## Electronic supplementary material

Below is the link to the electronic supplementary material.

Supplementary Material 1

Supplementary Material 2

## Data Availability

The data that support the findings of this study are available from Merative but restrictions apply to the availability of these data, which were used under license for the current study, and so are not publicly available. Data are, however, available from the authors upon reasonable request and with permission of Merative.

## Abbreviations

CDC: Centers for Disease Control and Prevention
ICD: International Classification of Diseases
IQR: Inter quartile range
MA: Modified algorithm
NNDSS: National Notifiable Disease Surveillance System
OA: Original algorithm
PPV: Positive predictive value
SD: Standard deviation
