# Supplementary material for: Optimizing identification of Lyme disease diagnoses in commercial insurance claims data, United States, 2016–2019
Source: BMC Infect Dis. 2024 Nov 20;24:1322. doi: 10.1186/s12879-024-10195-5 (PMC11580348; doi:10.1186/s12879-024-10195-5)
Supplement: Supplementary file 1 — Supplementary Material 1 [file 12879_2024_10195_MOESM1_ESM.docx]

Appendix A. Geographic risk level assigned to jurisdiction of residence^a^ in MarketScan database during 2016-2019.

| **High incidence^b^** |
| --- |
| Middle Atlantic Division, unknown state |
| New England Division, unknown state |
| Northeast Region, unknown division |
| Connecticut |
| Delaware |
| Maine |
| Maryland |
| Massachusetts |
| Minnesota |
| New Hampshire |
| New Jersey |
| New York |
| Pennsylvania |
| Rhode Island |
| Vermont |
| Virginia |
| Washington, DC |
| West Virginia |
| Wisconsin |
|  |
| **Unknown Incidence** |
| East North Central Division, unknown state |
| Nation, unknown region |
| North Central Region, unknown division |
| South Atlantic Division, unknown state |
| South Region, unknown division |
| West North Central Division, unknown state |
|  |
| **Low Incidence** |
| East South Central Division, unknown state |
| Mountain Division, unknown state |
| Pacific Division, unknown state |
| West Region, unknown division |
| West South Central Division, unknown state |
| Alabama |
| Alaska |
| Arizona |
| Arkansas |
| California |
| Colorado |
| Florida |
| Georgia |
| Hawaii |
| Idaho |
| Illinois |
| Indiana |
| Iowa |
| Kansas |
| Kentucky |
| Louisiana |
| Michigan |
| Mississippi |
| Missouri |
| Montana |
| Nebraska |
| Nevada |
| New Mexico |
| North Carolina |
| North Dakota |
| Ohio |
| Oklahoma |
| Oregon |
| Puerto Rico |
| South Carolina |
| South Dakota |
| Tennessee |
| Texas |
| Utah |
| Washington |
| Wyoming |

^a^For all patients where state or jurisdiction of residence was unknown but region or division was known, patients were assigned to geographic risk level based on the states or jurisdictions covered by the region or division. If all states or jurisdictions covered by the region or division were high incidence during the study period, they were assigned to the high incidence category. If all states or jurisdictions covered by the region or division were low incidence during the study period, they were assigned to the low incidence category. If states or jurisdictions covered by the region or division were a mix of high and low incidence during the study period, they were assigned to the unknown category.

^b^ High-incidence jurisdictions included those with ≥ 10 confirmed cases of Lyme disease per 100,000 population during the reporting period.
