# Supplementary material for: Optimizing identification of Lyme disease diagnoses in commercial insurance claims data, United States, 2016–2019
Source: BMC Infect Dis. 2024 Nov 20;24:1322. doi: 10.1186/s12879-024-10195-5 (PMC11580348; doi:10.1186/s12879-024-10195-5)
Supplement: Supplementary file 2 — Supplementary Material 2 [file 12879_2024_10195_MOESM2_ESM.pdf]

Appendix B. Days from diagnosis code to prescription claim among Lyme disease diagnoses in MarketScan database, 2016-2019.

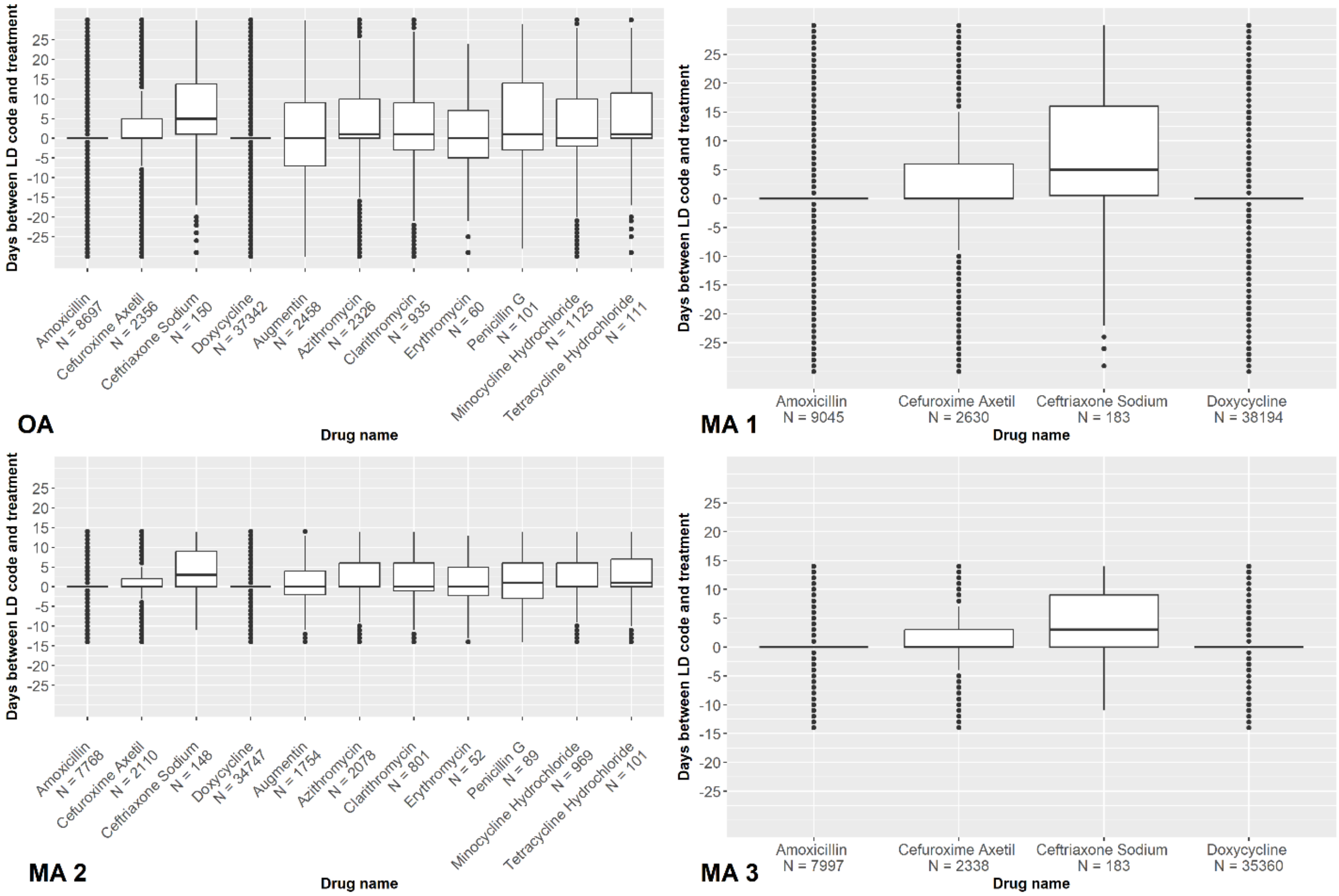

Legend: This plot displays data for qualifying prescription drugs according to each algorithm definition. OA = Original Algorithm; MA 1 = Modified Algorithm 1; MA 2 = Modified Algorithm 2; MA 3 = Modified Algorithm 3.
OA: Amoxicillin (Mean ± SD: 0.2 ± 9.0, Median: 0, IQR: 0); Cefuroxime axetil (Mean ± SD: 1.7 ± 10.2, Median: 0, IQR: 5); Ceftriaxone sodium (Mean: 6.5 ± 10.8, Median: 5, IQR: 12.8); Doxycycline (Mean ± SD: -0.7 ± 7.7, Median: 0, IQR: 0); Augmentin (Mean ± SD: 0.5 ± 14.4, Median: 0, IQR: 16); Azithromycin (Mean ± SD: 3.4 ± 12.4, Median: 1, IQR: 10); Clarithromycin (Mean ± SD: 2.4 ± 12.8, Median: 1, IQR: 12); Erythromycin (Mean ± SD: 0.8 ± 10.9, Median: 0, IQR: 12); Penicillin G (Mean ± SD: 4.1 ± 12.7, Median: 1, IQR: 17); Minocycline hydrochloride (Mean ± SD: 3.0 ± 12.0, Median: 0, IQR: 12); Tetracycline hydrochloride (Mean ± SD: 3.3 ± 12.0, Median: 1, IQR: 11.5)
MA 1: Amoxicillin (Mean ± SD: 0.3 ± 9.5, Median: 0, IQR: 0); Cefuroxime axetil (Mean ± SD: 2.2 ± 10.9, Median: 0, IQR: 6); Ceftriaxone sodium (Mean ± SD: 7.3 ± 11.8, Median: 5, IQR: 15.5); Doxycycline (Mean ± SD: -0.6 ± 8.0, Median: 0, IQR: 0)
MA 2: Amoxicillin (Mean ± SD: 0.1 ± 4.0, Median: 0, IQR: 0); Cefuroxime axetil (Mean ± SD: 1.1 ± 5.1, Median: 0, IQR: 2); Ceftriaxone sodium (Mean ± SD: 4.1 ± 5.8, Median: 3, IQR: 9); Doxycycline (Mean ± SD: -0.1 ± 3.9, Median: 0, IQR: 0); Augmentin (Mean ± SD: 0.5 ± 6.6, Median: 0, IQR: 6); Azithromycin (Mean ± SD: 1.5 ± 6.5, Median: 0, IQR: 6); Clarithromycin (Mean ± SD: 1.2 ± 6.8, Median: 0, IQR: 7); Erythromycin (Mean ± SD: 0.4 ± 6.8, Median: 0, IQR: 7.3); Penicillin G (Mean ± SD: 1.2 ± 6.9, Median: 1, IQR: 9); Minocycline hydrochloride (Mean ± SD: 1.1 ± 6.4, Median: 0, IQR: 6); Tetracycline hydrochloride (Mean ± SD: 2.1 ± 7.1, Median: 1, IQR: 7)
MA 3: Amoxicillin (Mean ± SD: 0.2 ± 4.2, Median: 0, IQR: 0); Cefuroxime axetil (Mean ± SD: 1.2 ± 5.3, Median: 0, IQR: 3); Ceftriaxone sodium (Mean ± SD: 4.0 ± 6.1, Median: 3, IQR: 9); Doxycycline (Mean ± SD: -0.1 ± 4.0, Median: 0, IQR: 0)
